# Multiple Cosmic Sources for Meteorite Macromolecules?

**DOI:** 10.1089/ast.2015.1331

**Published:** 2015-10-01

**Authors:** Mark A. Sephton, Jonathan S. Watson, William Meredith, Gordon D. Love, Iain Gilmour, Colin E. Snape

**Affiliations:** ^1^ Impacts and Astromaterials Research Centre, Department of Earth Science and Engineering, Imperial College London, London, UK.; ^2^ School of Chemical, Environmental and Mining Engineering, University of Nottingham, University Park, Nottingham, UK.; ^3^ Department of Earth Sciences, University of California, Riverside, California, USA.; ^4^ Centre for Earth, Planetary, Space and Astronomical Research, The Open University, Milton Keynes, UK.

## Abstract

The major organic component in carbonaceous meteorites is an organic macromolecular material. The Murchison macromolecular material comprises aromatic units connected by aliphatic and heteroatom-containing linkages or occluded within the wider structure. The macromolecular material source environment remains elusive. Traditionally, attempts to determine source have strived to identify a single environment. Here, we apply a highly efficient hydrogenolysis method to liberate units from the macromolecular material and use mass spectrometric techniques to determine their chemical structures and individual stable carbon isotope ratios. We confirm that the macromolecular material comprises a labile fraction with small aromatic units enriched in ^13^C and a refractory fraction made up of large aromatic units depleted in ^13^C. Our findings suggest that the macromolecular material may be derived from at least two separate environments. Compound-specific carbon isotope trends for aromatic compounds with carbon number may reflect mixing of the two sources. The story of the quantitatively dominant macromolecular material in meteorites appears to be made up of more than one chapter. Key Words: Abiotic organic synthesis—Carbonaceous chondrite—Cosmochemistry—Meteorites. Astrobiology 15, 779–786.

## 1. Introduction

In the early Solar System, the agglomeration of solid materials led to the formation of planetesimals and eventually the planets. Following accretion, solid materials were subjected to the action of heat and water, and their chemical and mineral constitution progressively changed (Krot *et al.*, 2006). Though these processes are consigned to history, the asteroids represent arrested stages in planet formation and as such are chronicles of these early Solar System events. Fragments of asteroids are naturally delivered to Earth as meteorites, providing accessible samples for study. Some of the most ancient records are found in the carbonaceous chondrites, which have elemental abundances that resemble the Sun and contain significant amounts of carbon (Weisberg *et al.*, 2006).

The majority of carbon in carbonaceous chondrites is present as organic matter that exhibits variability in chemical function and molecular weight. Trace amounts exist of low-molecular-weight compound classes such as aliphatic hydrocarbons, aromatic hydrocarbons, amino acids, nucleic acid bases, carboxylic acids, phosphonic acids, sulfonic acids, organohalogens, and polyhydroxylated compounds (Sephton, 2002). Yet by far the major organic component in meteorites is a large and complex organic network (Sephton *et al.*, 2000; Cody and Alexander, 2005; Alexander *et al.*, 2007) referred to by operational (insoluble organic matter or IOM) or descriptive (macromolecular material) terms. The macromolecular material is a complex network of aromatic units connected by aliphatic and heteroatom-containing linkages (Gardinier *et al.*, 2000; Cody *et al.*, 2002, 2011; Huang *et al.*, 2007). The macromolecular material is insoluble in common organic solvents and is generally assumed to be completely indigenous owing to its immobility. However, it is the intractability of the macromolecular material that creates analytical difficulties and repulses straightforward investigations (Sephton 2012).

The complexity of macromolecular material structure has been exposed by nondestructive spectroscopic techniques such as nuclear magnetic resonance (NMR) spectroscopy (*e.g.*, Gardinier *et al.*, 2000; Cody *et al.*, 2002) and X-ray absorption near-edge structure (XANES) spectroscopy (*e.g.*, Orthous-Daunay *et al.*, 2010), but to directly access the units from which macromolecular materials were constructed, degradative techniques must be employed. Perhaps the most commonly applied degradative methods have involved the use of pyrolysis, which thermally fragments the macromolecular material in an inert (or, more recently, hydrogen-contributing) atmosphere. Once liberated, macromolecular entities can be characterized, and their record of macromolecular material construction and subsequent processing can be read. The literature contains many pyrolysis-based studies of macromolecular material constitution and has been recently reviewed (Sephton, 2012).

The application of both pyrolysis and stable isotope methods has revealed genetic relationships between aromatic units in the macromolecular material and aromatic organic compounds found free in the meteorite (Sephton *et al.*, 1998). Liberated aromatic macromolecular entities often show structural and isotopic similarities to their free counterparts. Close inspection of the cases where stable carbon isotope data are available for both free and macromolecular compounds reveals a consistent enrichment in ^12^C in the free compounds. This indicates that the free aromatic compounds have been released from the macromolecular material in a pre-terrestrial degradation event (Sephton *et al.*, 1998).

Previous work on Murchison has identified a notable lower-molecular-weight (C_6_ to C_11_) trend of increasing ^13^C content with carbon number for free aromatic hydrocarbons (Sephton *et al.*, 1998; Sephton and Gilmour, 2001). This trend would be consistent with the production of simple compounds from the preferential cracking of ^12^C-containing bonds in a more complex starting material. The opposite trend was established for the free C_14_ to C_20_ polycyclic aromatic hydrocarbons (PAHs) in Murchison (Gilmour and Pillinger, 1994), consistent with the synthesis of higher-molecular-weight compounds from simpler precursors, which proceeds by the preferential addition of ^12^C as implied previously for other meteoritic compounds (Yuen *et al.*, 1984). The C_6_ to C_11_ trend of increasing ^13^C content with carbon number has also been established for aromatic hydrocarbons liberated from the macromolecular material by pyrolysis (Sephton *et al.*, 1998).

There is an increasing recognition that the macromolecular materials in meteorites are not single entities but can be subdivided into fractions with different thermal stabilities (Komiya *et al.*, 1993; Okumura and Mimura, 2011). Thermal degradation and stable isotope measurements reveal substantial isotopic heterogeneity (Halbout *et al.*, 1986; Kerridge *et al.*, 1987) and at least two operationally defined fractions (Sephton *et al.*, 2003, 2004). Labile organic matter is liberated by pyrolysis and is enriched in ^13^C and ^15^N, while refractory organic matter is relatively resistant to most pyrolysis techniques and is comparatively depleted in ^13^C and ^15^N (Sephton *et al.*, 2003, 2004). The recognition of two fractions in the meteorite macromolecular materials provokes a reassessment of previously recognized compound-specific stable carbon isotope patterns. If pyrolysis techniques can access both macromolecular fractions, a more complete picture of synthetic processes may be forthcoming.

Pyrolysis techniques vary in their ability to delve deeply into macromolecular material structure. Efficiency of pyrolysis methods for macromolecular materials can be assessed by measurement of experimental yields. Experiments on terrestrial kerogens indicate that hydropyrolysis, which refers to pyrolysis assisted by high hydrogen gas pressures and a dispersed catalyst, commonly converts more than 85% of the macromolecular structure to solvent-soluble hydrocarbons (*e.g.*, Roberts *et al.*, 1995). Hydropyrolysis targets C-O and C-S bonds below 400°C in the heating cycle but also cleaves C-C bonds at higher temperatures (Love *et al.*, 1995). The preeminence of catalytic hydrogenation methods for efficient pyrolysis of macromolecules is unsurprising when it is considered that pyrolysis methods are often hindered by hydrogen limitation and the formation of residues comprising hydrogen-poor char.

When applied to meteorites, hydropyrolysis appears to be the most efficient thermal degradation method for converting meteorite macromolecules to lower-molecular-weight fragments based on stable isotope analyses of experimental residues. The presence of two macromolecular fractions with distinct stable isotopic compositions allows mass balance calculations to be used as proxies for efficiency (Sephton *et al.*, 2004). Additional benefits from hydropyrolysis include minimal product rearrangements, thereby avoiding alteration of organic structures and stereochemistries (Love *et al.*, 1995). The products of catalytic hydrogenation are devoid of exocyclic heteroatom-containing groups and are highly amenable to analysis by gas chromatographic and mass spectrometric techniques.

It was our aim to use hydropyrolysis to provide an unprecedented level of access to aromatic units from both labile and refractory organic fractions within the complex organic network. We liberated organic units from deep within the macromolecular material in the Murchison meteorite to better understand its constitution and therefore origin. Our data reveal that meteorite macromolecular materials appear to contain a rich record of multiple cosmic processes.

## 2. Experimental

### 2.1. Sample preparation

Crushed whole meteorite (*ca.* 250 mg) was prepared for hydropyrolysis treatment by performing solvent extraction [ultrasonication with a solvent mixture of 95:5 dichloromethane (DCM)/methanol followed by removal of the supernatant, 4 mL ×3], a process that removes any free organic matter. The extraction protocol is in accord with previous hydropyrolysis work on terrestrial samples (Love *et al.*, 1995) and meteorite samples (Sephton *et al.*, 2004). Hydrogenation removes exocyclic heteroatoms (Sephton *et al.*, 2007; Ascough *et al.*, 2010) and will convert any residual water-soluble compounds such as amino acids and carboxylic acids to very light hydrocarbons, which are not retained for analysis. Similarly, any formaldehyde polymers as proposed for a source of meteorite macromolecular material (Cody *et al.*, 2011) are also likely to be invisible to the hydropyrolysis technique.

### 2.2. Hydropyrolysis

The sample was impregnated with an aqueous solution of ammonium dioxydithiomolybdate [(NH_4_)_2_MoO_2_S_2_] to give a nominal loading of molybdenum of 2 wt %. (NH_4_)_2_MoO_2_S_2_ reductively decomposes *in situ* under hydropyrolysis conditions above 250°C to form a catalytically active sulfided molybdenum phase. Hydropyrolysis runs were performed in an open-system, temperature-programmed reactor configuration, which has been described in detail previously (Love *et al.*, 1995). In this investigation, catalyst-loaded solvent-extracted sample was initially heated in a stainless steel (316 grade) reactor tube from ambient temperature to 220°C at 300°C min^−1^, then to 520°C at 8°C min^−1^, using a hydrogen pressure of 15 MPa. A constant hydrogen sweep gas flow of 6 dm^3^ min^−1^, measured at ambient temperature and pressure, through the reactor bed ensured that the residence times of volatiles generated from pyrolysis were extremely short, of the order of a few seconds. Although important volatiles are generated during pyrolysis experiments (Pizzarello *et al.*, 2011), the semi-volatile hydropyrolysis products (the hydropyrolysate) were the focus of this study, and these entities were collected on a silica trap that was cooled with dry ice (Meredith *et al.*, 2004; Sephton *et al.*, 2005). The hydropyrolysate was desorbed from the silica using DCM (*ca.* 4 mL), and elemental sulfur was removed with activated copper turnings. To reduce levels of background contamination, a cleaning run was performed prior to the sample run whereby the apparatus was heated to 520°C by using a rapid heating rate (300°C min^−1^) under high hydrogen pressure conditions.

### 2.3. Gas chromatography–mass spectrometry

Gas chromatography–mass spectrometry (GC-MS) analysis was carried out with an Agilent Technologies 6890 gas chromatograph coupled to a 5973 mass spectrometer. Separation was performed on a J&W DB-5MS column (30 m length, 0.25 mm internal diameter, and 0.25 μm film thickness) with a He carrier gas at a constant flow rate of 1.1 mL min^−1^. The gas chromatograph oven temperature was held for 10 min at 30°C and ramped to 300°C at a rate of 4°C min^−1^ and then held for 9 min. Peak identification was based on retention time and mass spectra comparisons with authenticated standards, well-characterized aromatic fractions of a coal tar and crude oil, and published reports (*e.g.*, Kruge, 2000, and references therein). In this paper, GC-MS data is presented as either total ion chromatograms (TICs), where the complete response of the extract is displayed, or partially reconstructed ion chromatograms.

### 2.4. Gas chromatography–combustion–isotope ratio mass spectrometry

Compound-specific δ^13^C analysis was carried out separately to GC-MS using a Thermo Scientific TraceUltra gas chromatograph fitted with a J&W DB-1 column (60 m length, 0.25 mm internal diameter, and 0.25 μm film thickness). Injection was splitless at 250°C with a constant column flow rate of 1.3 mL min^−1^.The gas chromatograph oven temperature was held for 4 min at 35°C and ramped to 310°C at a rate of 5°C min^−1^ and then held for 9 min. After combustion of the compounds and the removal of water, the CO_2_ was analyzed by a Thermo Scientific 253 isotope ratio mass spectrometer. The isotopic composition of individual compounds was measured against the pulses of calibrated CO_2_ reference gas at the start of the run. Only compounds that were present in abundance and that formed discrete peaks, free from problems of coelution, were measured for their individual stable isotopic compositions. Standards run on the gas chromatograph–mass spectrometer were also run under the same gas chromatograph conditions by gas chromatography–combustion–isotope ratio mass spectrometry (GC-C-IRMS) to determine retention times. The abundances of stable isotopes are expressed with the δ notation. These indicate the difference, in per mill (‰), between the relevant ratio in the sample and the same ratio in an international standard as follows: δ‰ = [(*R*_sample_ − *R*_standard_)/*R*_standard_] × 1000, where *R* = ^13^C/^12^C for carbon and the standard = PDB. Samples were run in triplicate, and all standard deviations were less than 0.3‰.

## 3. Results

**FIG. 1.**
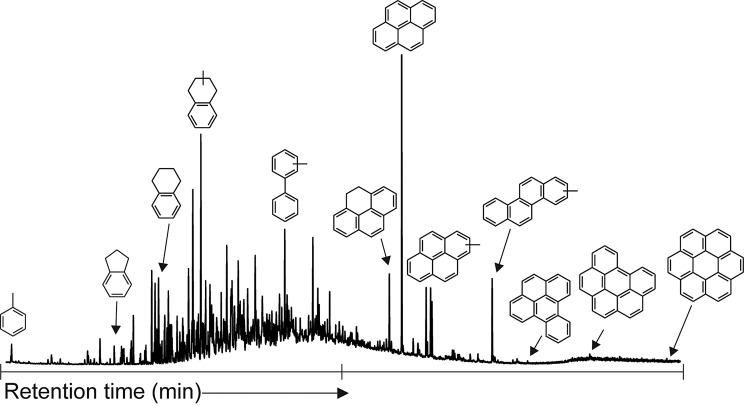
Total ion current of Murchison hydropyrolysis products. Some notable aromatic structures indicated.

**FIG. 2.**
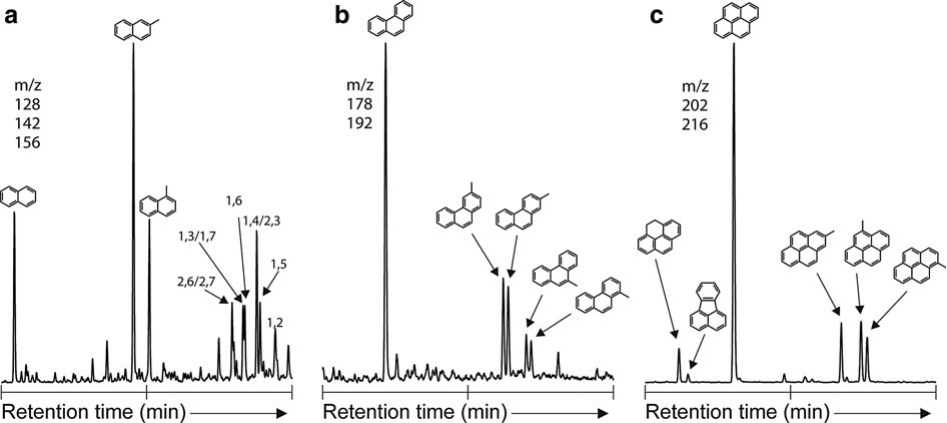
Partially reconstructed ion chromatograms from GC-MS analyses of Murchison hydropyrolysis products. Numbers indicate the position of methyl substituents. (**a**) the naphthalenes (*m/z* 128 + 142 + 156), (**b**) the phenanthrenes (*m/z* 178 + 192), and (**c**) the pyrenes (*m/z* 202 + 216) released upon hydropyrolysis of Murchison.

We released numerous one- to six-ring aromatic hydrocarbons and their methyl-substituted homologues. Utilizing a silica trap cooled with dry ice (Meredith *et al.*, 2004; Sephton *et al.*, 2005) allowed the retention of more volatile units compared to the earliest hydropyrolysis work on meteorites (Sephton *et al.*, 2004). Pyrene was the most abundant compound released during hydropyrolysis, as can be seen in the TIC (Fig. 1) where abundant parent PAHs are present. The hydropyrolysis products reveal some similarities to previous pyrolysis experiments that retained lower-molecular-weight methyl-substituted benzenes (Remusat *et al.*, 2005; Sephton *et al.*, 1998), and the relative distribution of substituted PAHs is also as would be expected from previous hydropyrolysis work (Fig. 2; Sephton *et al.*, 2004). An unresolved complex mixture of coeluting aromatic and aliphatic compounds underlies the discrete peaks in the TIC.

Owing to the great efficiency of hydropyrolysis for converting meteorite macromolecules to lower-molecular-weight fragments, both small one- and two-ring aromatic units and larger five- and six-ring PAHs are released from meteorite macromolecular materials in substantial amounts. It has been proven by pyrolysis studies that the labile organic matter is made up of mainly small aromatic units, and it has been predicted by the behavior of experimental residues that the refractory organic matter fraction may be made up of units containing three or more rings (Sephton *et al.*, 2004). Up to five- or six-ring entities have been generated from meteorite macromolecular materials before (Naraoka *et al.*, 2000; Sephton *et al.*, 2004; Yabuta *et al.*, 2007), but subsequent compound-specific isotope measurements have not been achieved for a wide molecular weight range of products. We obtained stable carbon isotope data for individual hydropyrolysis products ranging from one- to four-ring aromatic hydrocarbon units using GC-C-IRMS (Table 1).

**Table 1.** Organic Fractions Proposed for the Murchison Macromolecular Material

*Fraction*	*Bulk/mass balance δ^13^C‰*	*Compound specific δ^13^C‰*	*Organic structures*
Labile organic matter (LOM)	−5	−6.3 to −2.5	
Refractory organic matter (ROM)	−20	−25.5 to −20.5	

The stable carbon isotopic composition of proposed fractions obtained by mass balance measurements (Sephton *et al.*, 2003) are presented alongside corresponding structures and compound-specific isotope data for hydropyrolysis products from this study.

## 4. Discussion

High-resolution stepped combustion data indicate that labile organic matter is relatively enriched in ^13^C and ^15^N, while refractory organic matter is depleted in the same isotopes (Sephton *et al.*, 2003). It is possible that the individual low-molecular-weight compounds observed in our hydropyrolysis products represent the labile organic matter and the higher-molecular-weight units represent contributions from the refractory organic matter. A test for this hypothesis would be a correspondence of compound-specific isotope data for the smaller hydropyrolysis products with those suggested for labile organic matter (δ^13^C −5‰) and a similar stable isotopic match between the larger hydropyrolysis products with those postulated for the refractory organic matter (δ^13^C −20‰) (Sephton *et al.*, 2003). We note that confirmation of this concept would denote the first detection of purely organic signals from both fractions, because previous stepped combustion data contain a potentially wide mixture of combustible carbon-bearing components. Our data (Table 1) reveal that the carbon isotopic compositions for the smaller (C_8_ to C_10_; one- and two-ring) aromatic compounds are relatively enriched in ^13^C (δ^13^C −6.3 to −2.5‰), while the larger (C_16_ to C_19_; three- and four-ring) aromatic compounds released from the Murchison macromolecular material by hydropyrolysis are relatively depleted in ^13^C (δ^13^C −25.5 to −20.5‰). Notably, our carbon isotopic values for small and large aromatic hydropyrolysis products do approximate those postulated for the labile and refractory organic matter portions of the macromolecular material, respectively (Sephton *et al.*, 2003) (Table 1).

Two potential source environments for aromatic units within meteorite macromolecular materials are presolar environments (Sephton and Gilmour, 2000; Busemann *et al.*, 2006) and the protoplanetary disk (Morgan *et al.*, 1991; Remusat *et al.*, 2009). Although the ranges are large, presolar environments are characterized by ^13^C enrichments (Langer and Penzias, 1990) similar or greater than those seen in labile organic matter, while the Solar System is marked by relatively ^12^C-rich values (Clayton and Nittler, 2004) such as those seen in the refractory organic matter. These general source assignments are supported by previous stepped combustion studies that confirm temperature steps corresponding to the labile fraction (δD = 750 to 1264‰) are more D-rich than those for the refractory fraction (δD = 476 to 951‰) (Kerridge *et al.*, 1987); D-enrichments are a generally accepted indicator of presolar contributions (Wannier, 1980; Remusat *et al.*, 2006). Confirmed correlation of the relatively small and relatively large aromatic units from the macromolecular material with presolar and Solar System stable isotope ratios, respectively, suggests intriguing lines of further investigation.

The differences in the sizes of the aromatic units in the two fractions could be related to synthetic conditions in the two different environments. Furthermore, once the monomer units were formed, subsequent polymerization processes could have required certain conditions and specific reactants. Different polymerization reactions have the potential to produce characteristic organic linkages. Environments in which polymerization occurred may have very different conditions to those in which synthesis of the aromatic monomers took place. Hence, the relevant types and timings of polymerization mechanisms for aromatic units may also provide valuable constraints on possible source environments and any interposing events.

**FIG. 3.**
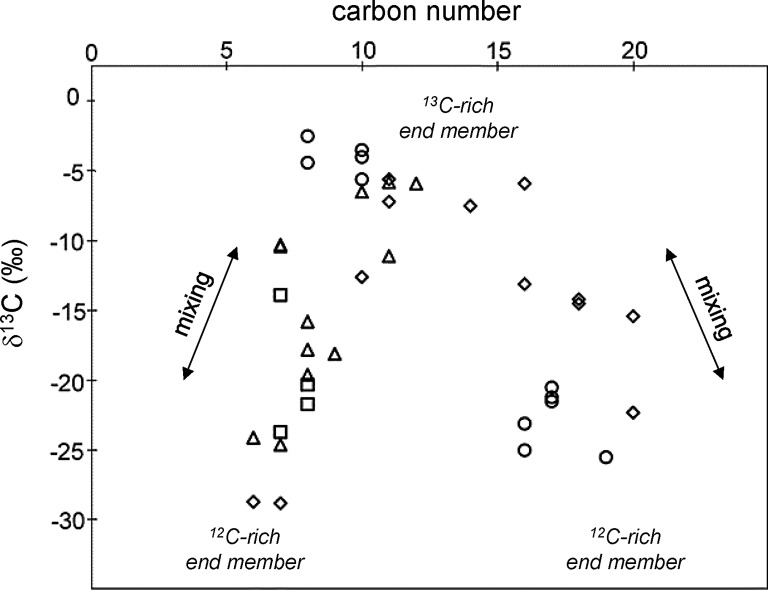
Carbon isotopic compositions (‰) of individual Murchison aromatic units plotted against carbon number. Symbols reflect different pyrolysis techniques (◊ = free compounds; □ = pyrolysis–gas chromatography–combustion–isotope ratio mass spectrometry; ○ = hydrous pyrolysis; ▵ = hydropyrolysis). One explanation for trends of δ^13^C with carbon number could be the mixing of two isotopically different but structurally overlapping components. Data are in Table 2.

In previous studies, workers have attempted to explain compound-specific carbon isotope data for macromolecular aromatic units by formation in a single environment (Sephton and Gilmour, 2000). The recognition of two isotopically distinct fractions in the macromolecular material provides new ways to interpret the combined data set of compound-specific carbon isotope values for free and macromolecular units (Table 2). The genetic relationship indicated by structural and isotopic similarities between free and macromolecular aromatic units (Sephton *et al.*, 1998) suggests that source apportionment for one fraction also applies to the other. A high-molecular-weight trend of decreasing ^13^C contents with carbon number (Fig. 3) may be explained as a mixing trend with labile and refractory organic matter end members. A recognized low-molecular-weight trend with increasing ^13^C contents with increasing carbon number in the C_5_ to C_10_ range (Fig. 3) could also represent a mixing trend if the very smallest aromatic units were produced concomitantly with the larger refractory organic matter units. Bimodal size distributions have been observed in some PAH formation mechanisms (Esarte *et al.*, 2009; Sánchez *et al.*, 2013). Mixing of two sources, therefore, could produce the observed trends of compound-specific carbon isotopes with carbon number.

**Table 2.** δ^13^C Values for Hydropyrolysis Products Alongside Other Free and Pyrolysis Data for Aromatic Units in Murchison

*Compound*	*# rings*	*# C*	*# subs*	*Free δ^13^C‰*	*Ref*	*PyGC-C-IRMS δ^13^C‰*	*Ref*	*H_2_O-Py δ^13^C‰*	*Ref*	*H_2_-Py δ^13^C‰*
benzene	1	6	0	−28.7 ± 0.2	[1]					
toluene	1	7	1	−28.8 ± 1.1	[2]	−1.3 ± 2.0	[3]	−24.6 ± 0.2	[2]	−6.3
						−5.4	[3]			
meta+para xylene	1	8	2					−19.6	[2]	−2.5
meta-xylene		8	2			−21.7 ± 0.4	[3]			
						−20.3	[3]			
ortho-xylene	1	8	2					−17.8	[2]	−4.4
C_3_-alkylbenzene	1	9	3					−18.1	[2]	
benzaldehyde	1	7	1			−23.7 ± 0.6	[3]			
						−24.0 ± 0.3	[3]			
phenol	1	6	1					−24.1	[2]	
2-methylphenol	1	7	2					−10.3	[2]	
3-methylphenol	1	7	2			−13.9 ± 0.9	[3]	−10.4	[2]	
	1					−18.5 ± 0.9	[3]			
indane	2	9	0							
5-methylindane	2	10	1							−5.6
4-methylindane	2	10	1							−3.5
tetralin	2	10	0							−4.0
naphthalene	2	10	0	−12.6 ± 2.3	[2]			−6.5	[2]	
benzothiophene	2	8	0					−15.8	[2]	
2-methylnaphthalene	2	11	1	−5.8	[2]			−5.6 ± 2.1	[2]	
1-methylnaphthalene	2	11	1	−11.1	[2]			−7.2 ± 2.0	[2]	
biphenyl	2	12	1							
acenaphthene	3	12	0					−5.9 ± 1.7	[2]	
phenanthrene	3	14	0	−7.5 ± 1.2	[4]					
4,5-dihydropyrene	3	16	0							−25.0
fluoranthene	4	16	0	−5.9 ± 1.1	[4]					
pyrene	4	16	0	−13.1 ± 1.3	[4]					−23.1
2-methylpyrene	4	17	1							−21.5
4-methylpyrene	4	17	1							−20.5
1-methylpyrene	4	17	1							−21.2
chrysene	4	18	0	−14.5 ± 2.2	[4]					
methylbenzo[c]phenanthrene	4	19	0							−25.5
benzo[ghi]fluoranthene	5	18	0	−14.2 ± 2.2	[4]					
benzo(e)pyrene	5	20	0	−22.3 ± 4.1	[4]					
benzo(j)fluoranthene	5	20	0	−15.4 ± 3.3	[4]					

References: [1] Yuen *et al.* (1984); [2] Sephton *et al.* (1998); [3] Sephton and Gilmour (2001); [4] Gilmour and Pillinger (1994).

# rings = number of rings; # C = carbon number; # subs = number of substituent carbon atoms on rings; H_2_O-Py = hydrous pyrolysis; H_2_-Py = hydropyrolysis.

Mixing of the two isotopically distinct labile and refractory organic matter fractions may not, however, be the only source of isotopic variation observed in pyrolysis products. Structurally identical aromatic units liberated from the labile organic matter fraction of the macromolecular material vary in their isotopic composition depending on how far the process of conversion from high-molecular-weight parent to low-molecular-weight fragments has proceeded (Sephton *et al.*, 1998). Owing to mass balance constraints, complete conversion of a macromolecular fraction will produce liberated aromatic units that reflect the overall stable isotopic composition of their parent fraction. Similarly, partial conversion can lead to liberated aromatic units that are less representative of the overall stable isotopic composition of their starting material. The isotopic fractionation brought about by partial conversion will depend on the isotopic heterogeneity of the units within the parent fraction. Moreover, previously published work has demonstrated that parent body alteration can convert the high-molecular-weight labile macromolecular organic fraction to low-molecular-weight fragments, so the isotopic values of free aromatic compounds and remaining labile macromolecular organic fraction can act as indicators of secondary processing (Sephton *et al.*, 1998, 2003).

It also follows that the efficiency of the pyrolysis technique used to access the labile organic matter fraction will also affect the stable isotopic values of the liberated units. Previously published work indicates that different pyrolysis techniques have produced structurally identical aromatic units from the macromolecular material with varying stable isotopic compositions (Table 2). If different pyrolysis techniques are assumed to reflect varying levels of efficiency of converting the labile organic fraction to low-molecular-weight products, and there is at least some evidence that this is the case (Sephton *et al.*, 2003, 2004), then the wide range in values observed would appear to reflect substantial isotopic heterogeneity. In our hydropyrolysis data, we assume that the low-molecular-weight compounds reflecting the labile organic matter fraction represent complete conversion of their high-molecular-weight parent fraction, and hence both liberated units and parent labile organic matter fraction have concordant isotopic compositions. The isotopic values for the higher-molecular-weight hydropyrolysis products that represent the refractory organic matter are unlikely to represent complete conversion of the fraction, but parent and product values correspond owing to the isotopic homogeneity of the high-molecular-weight starting material. Stepped combustion data have indicated that the labile organic matter fraction is isotopically heterogeneous, while the refractory organic matter is isotopically homogeneous (Sephton *et al.*, 2003, 2004).

The recognition of possible multiple source environments for Murchison macromolecular materials suggests future research directions. Distribution of the two macromolecular fractions across meteorite types can reveal how parent bodies sampled and preserved presolar and protoplanetary disk organic inventories. Moreover, it is recognized that macromolecular materials in meteorites are associated with exotic grains (Bernatowicz *et al.*, 2006). The recognition of two distinct macromolecular fractions allows the hosting of exotic grains to be reinvestigated. Environmental and temporal constraints on events that led to the origin and juxtaposition of grains and their possible organic hosts can now be sought.

## 5. Conclusions

Hydropyrolysis was used to liberate units from more than one fraction of the macromolecular material in Murchison. The labile fraction produced small aromatic units enriched in ^13^C, while the refractory fraction contributed large aromatic units depleted in ^13^C. Compound-specific carbon isotope trends for aromatic compounds with carbon number may reflect mixing of the two sources. The relative timing of the various steps in macromolecular materials construction represents a rich record of cosmic chemistry.

## Abbreviations Used

DCM: dichloromethane
GC-C-IRMS: gas chromatography–combustion–isotope ratio mass spectrometry
GC-MS: gas chromatography–mass spectrometry
PAHs: polycyclic aromatic hydrocarbons
TIC: total ion chromatogram
